# Patient-reported outcome survey of user-experiences in the spinal cord injured-community with MPPT for treating wounds and pressure injuries and for controlling soft tissue infection caused by osteomyelitis

**DOI:** 10.3389/fresc.2024.1386518

**Published:** 2024-06-20

**Authors:** Damian Smith, Mark Ridler

**Affiliations:** Spinal Injuries Association, Milton Keynes, United Kingdom

**Keywords:** spinal cord injury, pressure ulcers, osteomyelitis, MPPT, telemedicine, wound, musculoskeletal, rehabilitation

## Abstract

**Background:**

People with spinal cord injury (SCI) are at high risk of developing pressure injuries. Reports in the SCI-community had indicated that a new class of wound treatment, MPPT (micropore-particle-technology), was effective in treating pressure injuries. The British Spinal Injuries Association therefore conducted a survey among MPPT-users to learn from their experiences.

**Methods:**

Online survey restricted to individuals with spinal cord injury. Participants were requested to identify themselves to permit validation of statement.

**Results:**

The survey had 41 respondents reporting on a total of 49 wounds of which the two main categories were wounds (*n* = 33), primarily pelvic pressure ulcers; and draining fistulas (*n* = 9) caused by osteomyelitis. All wounds reported had reached full closure. Median duration of MPPT use and time to closure were 3 and 4 weeks for acute wounds (<6 weeks old) and 8 and 10 weeks for chronic wounds, respectively. On draining fistulas, MPPT had been used to reduce wound size, remove soft tissue infection, avoid sepsis, reduce autonomic dysreflexia, improve overall health, and avoid bed rest, whilst waiting for surgery. Comments on MPPT were 84% highly positive, 11% positive, and 0% negative. No adverse events were reported.

**Conclusions:**

MPPT achieved a 100% closure rate of acute and chronic wounds, and, in draining fistulas, effectively controlled soft tissue infection resulting from osteomyelitis. MPPT does not require bed rest and is suitable for self-care and telemedicine, promoting independence and higher quality-of-life. The findings strongly agree with a recent clinical study of MPPT.

## Introduction

Spinal cord injury results in loss of communication between the nervous and the immune system, leading to immunosuppression (1–5). The consequences include an increased risk of developing pressure injuries as well as impaired ability to heal wounds and reduced ability to fight infection (6). As a result, the pressure ulcers frequently deteriorate and lead to the development of osteomyelitis (7, 8). Osteomyelitis more than 6-week-old can only be removed by surgery (9, 10). In a retrospective study of the outcome of surgery for pressure ulcer induced osteomyelitis in the pelvic area, Russell et al. (8) reported a failure rate of 71% of surgery and a median survival time of 2 years following first surgery if the wound post-surgery did not close (64% of cases) and 7 years if it did (36% of cases). Diagnostic tools such as x-ray, MRI and biopsies are not fully predictive of osteomyelitis and many consequently go undiagnosed (9–11). A typical sign is a non-healing wound, which in reality is a draining fistula through which infectious debris travels from the infected bone to the skin surface. These frequently cause extensive soft tissue infection associated with high levels of exudate, malodour, risk of sepsis, and increased frequency and severity of autonomic dysreflexia. Russell et al. (8) reported that the median age of a pressure ulcer causing osteomyelitis was 4 months and it could be as little as 6 weeks. Others have reported osteomyelitis within 2 weeks from wound detection (7). Pressure injuries resulting in osteomyelitis are therefore life-changing and often life-terminating with 10%–12% of SCI-persons dying from pressure ulcers (12–14). Since the available wound products are ineffective (15, 16), SCI-persons live in constant fear of pressure injuries.

SIA (Spinal Injuries Association) is a national charity working on behalf of spinal cord injured people in England, Wales, and Northern Ireland. Among its members, a new product class, MPPT (technical name: micropore particle technology; tradename Amicapsil-SCI®, manufactured by Willingsford Ltd., Southampton, UK) has attracted interest for the treatment of wounds and pressure ulcers and for controlling the consequences of osteomyelitis on soft tissue. MPPT is a CE-marked medical device, which is approved as a treatment for wounds, i.e., with a therapeutic claim, and for use in immunocompromised patients, including spinal cord injured. It uses physical forces, in the form of capillary-evaporation, to remove microbial toxins and to break up biofilm (17). The removal of the toxins enables the immune cells to regain their function and the disruption of the biofilm disables the shield protecting the bacteria, thereby rendering them exposed to the body's immune system, which has now become functional again. MPPT challenges the status quo of common practice in the management of wounds. Antimicrobial products are often used to fight “wound infection” or “wound colonisation”. However, antimicrobial agents such as antibiotics and antiseptics (e.g., silver, chlorhexidine, PHMB, etc.) kill the microbes without distinguishing between which ones are too high or too low in numbers to uphold the required microbial balance and whether their presence in the microbiome may actually be essential for healing. Recent findings have confirmed that the commensals, i.e., bacteria living naturally on the skin, are necessary for healing and that antimicrobial effects impair healing (18). Already in 2016, NICE (19) and the US-FDA (20), independently of each other, concluded that antimicrobials do not remove wound infections or promote wound healing. Furthermore, an increasing body of data report severe long-term complications from antibiotic use, including cancer, diabetes, mental health issues and foetal malformations (21–24). This questions their use in wound care when they do not provide strong clinical benefits.

MPPT represents a very new and different approach to wound treatment, which has been shown effective across wound types (17). The SCI-community is often quick to implement new approaches and technologies, and considerable benefits of MPPT for the treatment of pressure ulcers have been reported. SIA therefore decided to conduct a survey among MPPT-users in the SCI-community to learn from their experiences.

## Methods

The survey was a retrospective service evaluation designed to evaluate the experiences members of the SCI-community had with the use of MPPT. Service evaluations do not require ethical approval. The online survey was designed with 15 open- and closed-ended questions; the questions were designed in a manner that would not cause distress or pose a risk to the participants. It was advertised in a monthly newsletter sent to all SIA members and supporters by e-mail, on a number of closed SCI-focused Facebook groups, and by word of mouth. The survey was open for 4 weeks, July 8th to August 6th, 2022. Participants were informed that data would be anonymized, and that data would be stored according to GDPR regulations. It was a public survey and participation was voluntary. Responders were required to submit their full name to allow verification of the information, if needed. After removing all names, the responses were shared with the persons, who had been guiding many in the use of MPPT. The verification process led to two changes: one outcome was registered as stopping the use of MPPT prematurely and one as possible osteomyelitis. The former respondent stated that the first, but not the second bottle had worked. It is known that MPPT is temperature sensitive, and this took place during the summer period. Also, the person had expressed concern about having to perform dressing changes without nurse participation and that was the reason for stopping. The latter respondent submitted incorrect treatment information. Information on their wounds were retained in the survey.

MPPT is a powder that is applied topically to the wound. In persons with SCI, daily application is needed until closure due to their immunocompromised status. Once daily, the wound is washed thoroughly with tap-water, and MPPT is applied to all wound surfaces and all affected skin surrounding the wound. The wound can be left uncovered or covered with a single, woven, 100% cotton gauze swab, i.e., the wound is not occluded, but can easily breathe and evaporate moisture. MPPT requires access of air to function and, if air access to the wound surface is blocked, e.g., when sitting in a chair or applying an occlusive dressing, air can be provided via an air-pump. The bottles are not single use. MPPT is temperature sensitive and must be stored on refrigeration.

## Results

41 respondents completed the survey providing information on 49 wounds. 85% of the respondents had learned of MPPT via the UK SCI-community, i.e., 39% had heard about MPPT via word-of-mouth, e.g., friends, other SCI people and other wheelchair users; 29% from SIA; and 17% via closed SCI-community Facebook groups. 10% had had MPPT recommended by their nurse. 5% had heard about it from other SCI-patient organizations (Scotland and Denmark). The majority (38 respondents) were self-paying for MPPT, but 3 persons with uncontrolled draining fistulas had received exceptional funding from the National Health Service (NHS), which funds access to novel treatments.

### Type of wound

**Figure 1:**
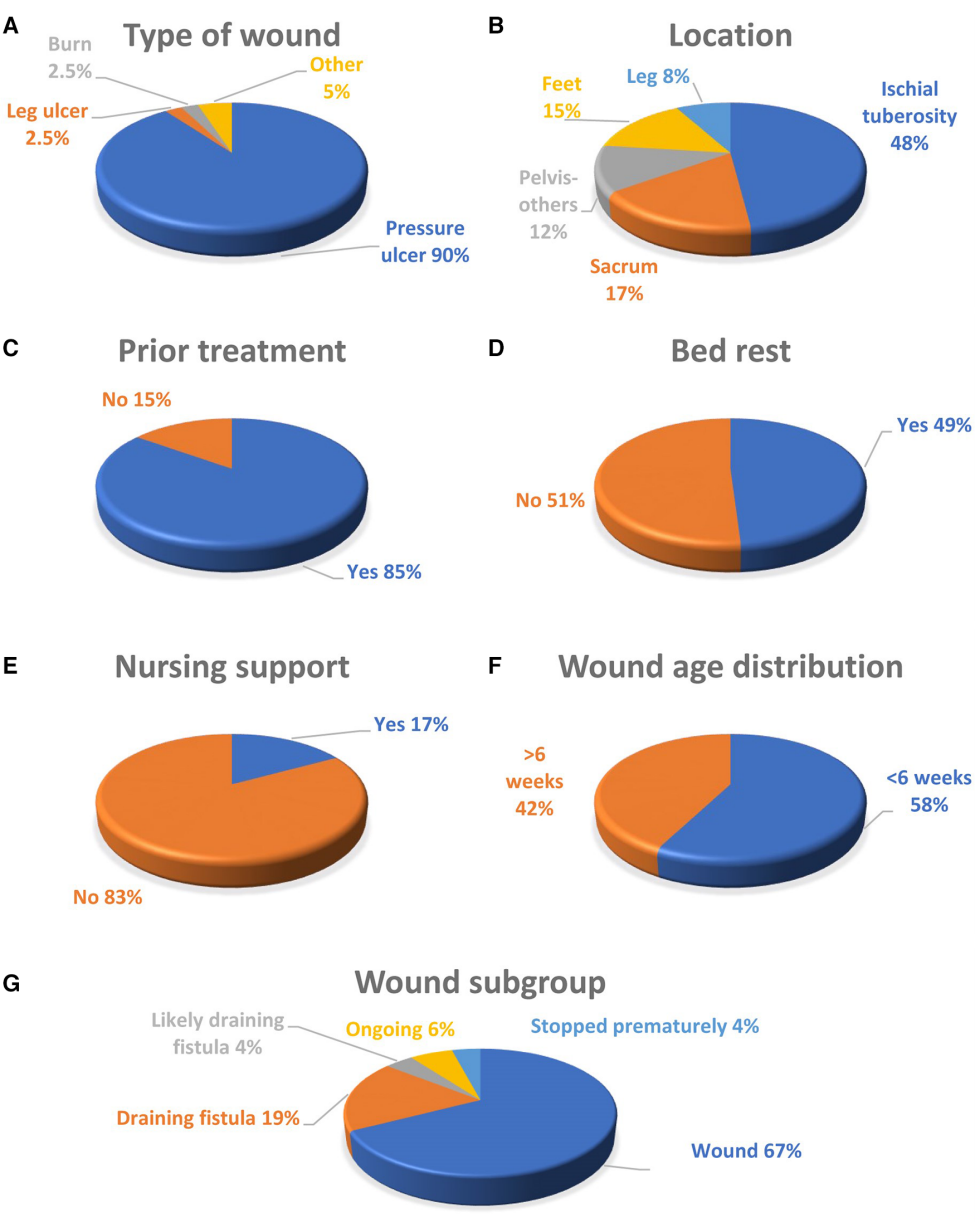
(**A**) The type of wound; (**B**) the location of the wound; (**C**) whether the wound had received care using standard wound products before changing to MPPT; (**D**) distribution of acute (<6 weeks old) vs. chronic (>6 weeks old) wounds; (**E**) whether the person had been on bed rest whilst using MPPT; (**F**) whether the respondents received nursing support with using MPPT; and (**G**) wound subgroups.

The wounds were primarily pressure ulcers (Figure 1A) with 77% in the pelvic region (Figure 1B), mainly on the ischial tuberosity and the sacrum, and 23% on legs and feet. 85% of wounds had been treated with other products before changing to MPPT (Figure 1C), indicating that these products had failed to promote closure. The types of products used included absorbent dressings, hydrogels, alginates, antimicrobials, e.g., Manuka honey, iodine, silver, antibacterial enzymes, and PHMB; and negative pressure wound therapy (NPWT) (see Supplementary S1 for details). Many stated they had tried “everything the NHS could offer”. 15% had used MPPT as first line of treatment.

### Outcome

The wound subgroups (Figure 1G) were 67% (*n* = 33) closed wounds; 19% (*n* = 9) draining fistulas; 6% (*n* = 3) wounds recently started and still undergoing treatment with MPPT; 4% (*n* = 2) likely draining fistulas based on comments and wound history; and 4% (*n* = 2) stopping treatment too early to experience a definitive outcome, one due to costs and one due to lack of efficacy after exposing the MPPT to heat and being unable to obtain nurse support. 49% of the respondents had been on bed rest whilst using MPPT (Figure 1D). The majority (83%) did not have nursing support whilst using MPPT for the treatment of their wounds, (Figure 1E).

For wounds and draining fistulas, the expected outcome, if using an effective wound treatment, is closure and control of soft tissue infection, respectively. Similar predictions cannot be made for the remaining wound subgroups. Therefore, to evaluate the efficacy of MPPT, the closure rate of wounds and the ability to control soft tissue infection associated with draining fistulas can be used as outcome measures (Table 1). The wounds were divided into acute and chronic subgroups, using 6 weeks as the dividing line (Figure 1F). The median age of acute wounds was 1.5 weeks compared to 19 weeks for chronic wounds (Table 1). The draining fistulas were considerably older with a median age of 2 years.

**Table 1:** Wound age, duration of use of MPPT and time to closure for acute and chronic wounds and draining fistulas caused by osteomyelitis.

*N*		Acute wounds <6 weeks old	Chronic wounds >6 weeks old	Draining fistula
		19	14	9
Age of wound	Mean ± SD	1.8 ± 1.7 weeks	36.1 ± 52.0 weeks	3.3 ± 3.1 years
	Median	1.5 weeks	19 weeks	2 years
Duration of use	Mean ± SD	5.3 ± 4.8 weeks	11.0 ± 9.0 weeks	1.4 ± 0.9 years
	Median	3 weeks	8 weeks	1.5 years
Time to closure	Mean ± SD	5.3 ± 4.6 weeks	12.7 ± 11.6 weeks	NA
	Median	4 weeks	10 weeks	

**Figure 2:**
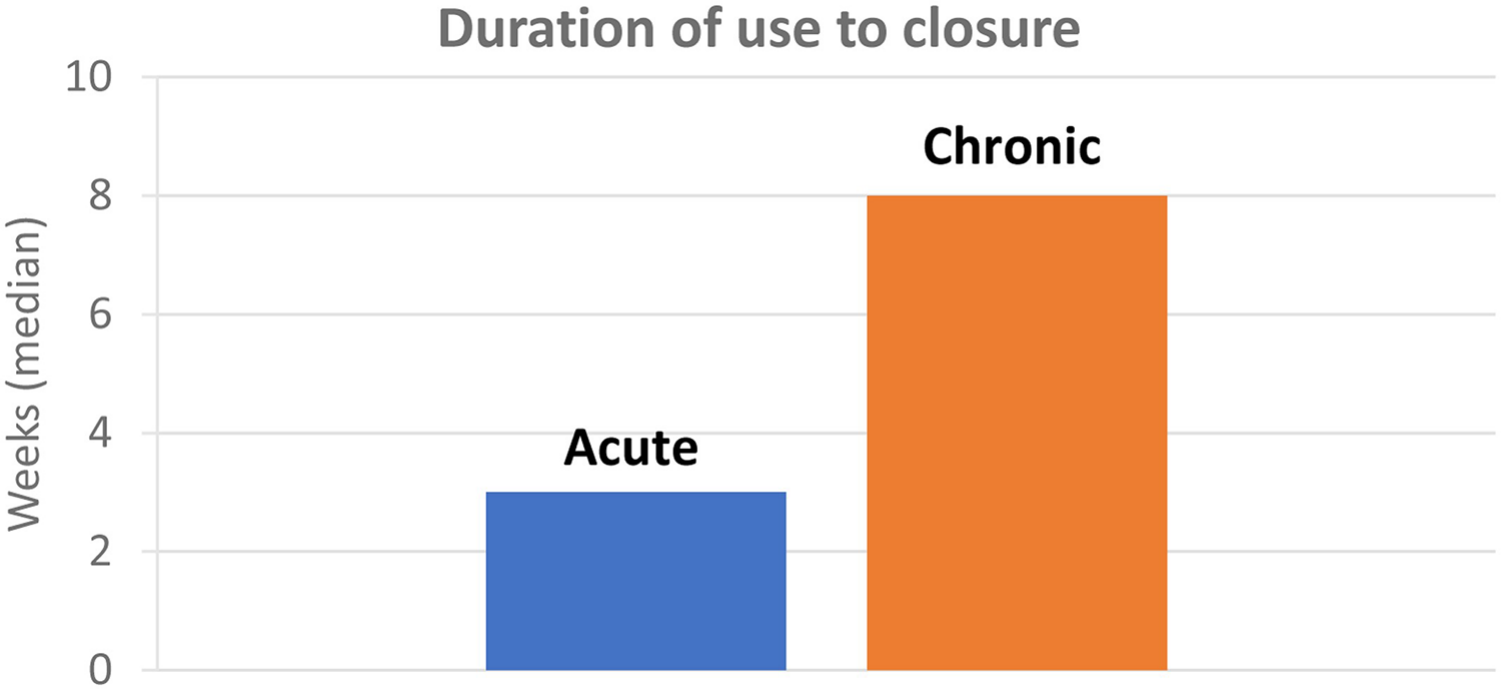
Median number of weeks of use of MPPT to achieve wound closure for acute (<6 week old) and chronic (>6 weeks old) wounds.

Treatment outcome was stable closure for all acute and chronic wounds, i.e., a 100% closure rate. The duration of use of MPPT to achieve closure (Figure 2) depended on the age of the wound at the start of MPPT treatment with a median of 3 and 8 weeks, respectively, for acute and chronic wounds; median time to closure were 4 weeks and 10 weeks, respectively. For draining fistulas, which can only close after surgically removing the underlying osteomyelitis, the median time respondents had used MPPT was 1.5 years. The reasons given for continued use included reduced wound size, keeping the wound free of infection, avoiding sepsis, reduced autonomic dysreflexia, overall improved health, and avoiding bed rest. In 8 of 9 cases, MPPT was used daily. In the remaining case, it was used intermittently when family was able to help because nurse support was not available and the respondent could not get to the wound unsupported.

37 respondents provided comments on their use of MPPT (Table 2). 84% (*n* = 31) were highly positive; 11% (*n* = 4) positive; and 0% (*n* = 0) negative or very negative. 5% (*n* = 2) were uncertain whether the closure of their wounds was due to MPPT or to the use of water and access to air for treating the wound instead of standard care procedures. However, it is known that healing is not supported if the MPPT has been damaged by heat exposure, demonstrating that it is required for healing to take place. Also, because MPPT acts by supporting the immune system, its effects persist for some weeks after terminating treatment. There were no indications of MPPT having caused adverse events, even following daily use for up to 3 years. MPPT was generally considered easy to use and suitable for self-care.

**Table 2:** Answers provided by respondents when asked about their personal impression and experience with MPPT.

Wound category	Rate	Comments
Wound	HP	GOD SEND……
Wound	HP	Very effective. So easy to use and really works
Wound	HP	It was so good I just can't speak highly enough when all else had failed.
Wound	HP	Excellent experience. So easy to use and extremely effective.
Wound	HP	It was really good
Wound	HP	Very effective
Wound	HP	Very. Saw faster progress compared to traditional dressings once started
Wound	HP	I found Amicapsil to be very effective and healed the wound quickly
Wound	HP	Extremely effective vs. all other treatments tried. It has absolutely changed my life.
Wound	HP	Very effective
Wound	HP	Amazing!!! This stuff is a game changer!!! I suffer regularly from pressure sores, I’ve finally found something which aids healing
Wound	HP	The best dressing on the market. It is my “go to” treatment. I keep one in my fridge for emergencies.
Wound	HP	Excellent, works much quicker than other products resulting in less time confined to bed.
Wound	HP	Where I've used it on new wounds at the first sign of damage I'm very happy with its effectiveness.
Wound	HP	It's the best product I have ever used. I have had a few pressure sores. One resulting in being in bed for 4 and a half months. I would not hesitate to recommend this product to other people
Wound	HP	The Amicapsil along with detailed instructions were brilliant. The pressure ulcer has now healed properly and the skin has stayed intact for over 4 years now when it used to break down every few months.
Wound	HP	I’ve used it several times and in the worst affected it halved my bed rest time which was incredible and the main thing that can affect mental health. I’m prone to skin breakdowns and now wouldn't use anything else even when I don't get the support from healthcare providers.
Wound	HP	It did all the difference. Amicapsil together with the daily follow-up and relief made the wound heal. It was visible throughout the process, which had a positive effect on the mood, and action in relation to the tasks that had to be done from the bed during that period
Wound	HP	I had a similar burn but on my R thigh in 2007 - pre menopause and before T2D. It was treated traditionally, got infected 3times and broke down twice once healed. It wasn't as big or quite as deep as the one on my R thigh. I had no infections using Amicapsil & it didn't break down once healed. It was slow to heal initially because an esker had formed & local nurses would not remove it so that took almost 3 wks. Amicapsil also slowed when I had some ABs for a UTIband when I had a covid booster. Had it not been for these delays then the burn would have healed much quicker. I was advised beforehand by Willingsford Health that ABs, covid booster & esker would delay healing.
Wound	HP	This was my fourth pressure ulcer. I've had two - one on each side near the ischium - that tunnelled and got infected and need flap surgeries to close. The third sore was more on the glute/buttock and that healed on it's own, by cover with a hydrocolloid dressing. Amicapsil healed my 2021 left ischial pressure sore without necessitating surgery, antibiotics for infection, or using hydrocolloid dressings. I will say that I did immediately start spending more time on bed rest than I did with previous sores.
Wound	P	Good
Wound	P	Amicapsil was effective at treating my pressure wound. Unfortunately the position of the wound on my back did make the application and maintenance of it challenging (effectively because the powder is applied dry it was challenging to keep it *in situ* without lying prone). However, overall my experience was positive.
Wound	U	I do not know. The wounds improved significantly during the Amicapsil treatment but I think this was because of good advice on debriding and dressing the wound rather than the Amicapsil. I stopped Amicapsil after approx. 2 months but the wounds continued to heal.
Wound	U	Very difficult to quantify and to be clinically precise that this was the difference or not. It is not an immediate response and like all wound treatment it takes time, care and diligence to facilitate healing. I believe it helped with keeping an open wound clean and also the skin granulation, ke
Draining fistula	HP	Amicapsil is giving me an opportunity to get up every day, and therefore helps my respiratory function. Thanks to Amicapsil, the severity and frequency of AD episodes got reduced.
Draining fistula	HP	It has made my wound smaller and helped the surrounding skin which was very red causing autonomic dyslexia, which has reduced. I’m now to able to sit up during the day without headaches and sweating from being autonomic.
Draining fistula	HP	It has been invaluable to not getting sepsis whilst waiting for bone debridement surgery. The wound has closed by nearly 90% as still needs surgery
Draining fistula	HP	Amicapsil is amazing I am still using it on my left hip … my wound as decreased in size a good 60% … since using I have been intravenous free, hospital free, no osteomyelitis flare ups, no sepsis, healthier, no more antibiotics, infection free … Amicapsil is amazing and should be made available through the NHS
Draining fistula	HP	Since starting to use Amicapsil my sacral wound has improved greatly and has nearly healed on a couple of occasions. Because I have suspected osteomyelitis the exudate from the infected bone has to have a pathway out thus the wound will not completely heal until corrective surgery takes place, which is scheduled to happen at Sheffield next week.
Draining fistula	HP	Very good. SUMMARY PRE-SURGERY: Amicapsil arrested further deterioration in ulcer size, slightly reducing what was already a chronic wound; it very significantly moderated infection and exudate and kept tissue healthy; it allowed time away from bedrest while awaiting surgery; it allowed self administration of treatment. Personal circumstances forced a suspension of treatment for 2wks and then significant re-infection, antibiotics and exudate. POST-SURGERY UPDATE: In hospital now following surgery and a successful intervention. The consultant mentioned that my ulcer was “unusual” having multiple internal fistula channels rather than an unconstrained tissue breakdown (beyond that observed before starting Amicapsil); it is possible this is a feature from using Amicapsil although further case studies are needed
Draining fistula	HP	Very effective - partially healed wound and kept infection free until surgery
Draining fistula	HP	Unfortunately the wound was to old and advanced before starting treatment, but Amicapsil kept the wound and the surrounding skin clean and infection free up until it was closed surgically, April 2022
Draining fistula	HP	It was extremely effective but I could not afford to keep buying it. I didn't around £1,500 of my own money
Draining fistula	P	Amicapsil cleared all the dressing dermatitis. Kept wound clean but because osteomyelitis was diagnosed by an MRI was prevented from working
Likely draining fistula	HP	I think its the best treatment but its hard to apply it myself
Ongoing	HP	One of the best treatments I have found. Had made a massive difference in a small amount of time. Can't believe I’ve not used before and it's not recommended or available on NHS
Ongoing	P	pretty effective at ending the infection and assisting the healing process

HP, highly positive (84%); P, positive (11%); N, negative (0%); U, uncertain (5%) whether closure was due to MPPT or change in treatment procedures.

## Discussion

The survey reported a 100% closure rate of wounds with MPPT; median duration of use was 3 weeks for acute and 8 weeks for chronic wounds. All respondents with a draining fistula caused by osteomyelitis had used MPPT to reduce wound size, risk of sepsis, frequency and severity of autonomic dysreflexia, improve well-being, and avoid bed rest. None of the respondents mentioned having experienced any adverse events. MPPT had supported independence as 51% had not been on bed rest and 83% had managed their wound without nursing support, either themselves or with the help of family and carers. The comments on the use of MPPT were very positive, highlighting its efficacy, speed of improvement, and ease of use. SCI-persons are a group of people, who regularly develop pressure ulcers and who consequently become very familiar with available wound products, e.g., several wrote that “everything the NHS could offer” to treat their wound had been tried unsuccessfully before changing to MPPT.

The frequency of wounds and pressure injuries can be reduced through prevention, but they are not fully preventable. The impact of SCI on skin function and the fact that it causes impairment of the immune response will invariably result in the development of pressure injuries. Similarly, scratches to the skin will happen, e.g., during the frequent transfers, which can rapidly develop into a wound requiring treatment.

The risk of wounds leading to osteomyelitis is high, e.g., Rennert et al. (7) reported that 32% of grade 4 pressure ulcers resulted in osteomyelitis and this can happen in as little as 2 weeks. It is therefore vital to initiate effective treatment as soon as possible. However, NICE (15) has concluded that antibiotics, antiseptics, and NPWT should not be used for treating pressure ulcers and the US FDA (16) recently concluded that wounds not healing spontaneously constitute an unmet medical need due to lack of effective treatments; the FDA statement referred to all types of wounds and not only in SCI. Due to the lack of effective treatments, resulting in wounds frequently giving rise to osteomyelitis, pressure injuries have been estimated to be responsible for 25% of all healthcare costs of SCI-persons (25).

At the individual level, living with a non-healing wound over a period of weeks, months, or years will have significant impact on a person's quality of life, including mental health. This is both due to the constant fear of the wound deteriorating to cause osteomyelitis, or, if osteomyelitis is present, the fear of sepsis as well as the immediate consequences of the wound or draining fistula, e.g., high exudate levels, malodour, once or twice daily dressing changes, which may require cancelling activities and waiting at home for the visit by the nurse, and, in most cases, bed rest to support healing, sometimes for months to years. Additionally, the presence of infection in the body will cause toxaemia and impact well-being as well as physical strength. Life itself ends up revolving around the wound, and external necessary activities and responsibilities, such as holding a job, running a business, being a parent, or taking an education become increasingly difficult to carry out and fulfil. It also interferes with the ability to be involved in rehabilitation activities, making it even more difficult to rebuild a life (26, 27). These consequences gradually lead to increased isolation and unavoidably wear down the person's mental health and quality of life.

The MPPT treatment process involves daily washing of the wound with tap-water, application of the MPPT-powder to all wound surfaces, and either dressing with a woven, pure cotton gauze swab or leaving the powdered wound uncovered. Remote assistance in the use of MPPT is available from the manufacturer, and this allows people themselves to be responsible for their wound. In the survey, 83% did not receive nurse-support and they achieved good outcomes as shown by the responses. As MPPT works via evaporation, the wound surface needs to be exposed to air, either directly or by providing a very gentle airflow across the wound surface using a small air-pump. There is, however, no requirement for bed rest if air can be provided, allowing over half of the respondents to avoid this; extended bed rest is known to be detrimental to health (28, 29). The combination of self-care for the wound and no requirement of bed rest means that MPPT-users are free to schedule their own day and carry out required activities, including away from home, as they do not need to wait at home for the nurse to have their dressing changed. They can therefore lead an active life. This is particularly important when osteomyelitis is present as the waiting times for surgery can be prolonged (years) and some are inoperable. Furthermore, by learning how to use MPPT, treatment of new wounds can be initiated rapidly and with an increasing degree of independence, thereby reducing demand on the health service.

The novel mode-of-action of MPPT, which does not involve antimicrobials, allows it to be effective on antimicrobial-resistant infections and not to contribute to the development of new antimicrobial resistance. Furthermore, this avoids the adverse effects of antimicrobials, which newer studies have found to include cancer, diabetes, asthma, mental health issues, and foetal malformations. Finally, the components of MPPT are natural and biologically recyclable, and no chemicals or plastics are used in the treatment process, making it environmentally sustainable.

MPPT has previously, in a 266-patient RCT, been shown to remove wound infections 60% quicker and to initiate tissue regeneration 50% quicker than antibiotics and antiseptics across a range of acute wounds, diabetic foot ulcers, venous leg ulcers and burns (30). In a systematic case-series of dehisced surgical wounds in hospital in-patients, it was found to achieve a non-infected, healing wound suitable for discharge in 3–5 days whereas standard care normally required 1 week with a desloughing agent followed by 2 weeks or more with NPWT to reach the same healing stage (31). In persons with SCI, two case-reports reported substantial benefits of MPPT compared to chlorhexidine, Manuka honey, and a combination of PHMB antimicrobial and betaine surfactant (26, 32). A non-interventional, real-world-evidence, clinical study in persons with SCI found closure rates of 100% for acute and chronic wounds and pressure injuries and effective control of soft tissue infection in draining fistulas (33). This clinical study compared costs of treatment with MPPT to standard care and found total per wound cost savings in the range of 74%–89% for acute wounds and pressure injuries the first year and 100% the following years, because the wounds had closed. Nursing resources were freed up at comparable levels.

The present survey is based on patient-reported outcomes. This represents real-world-evidence provided by patients without the involvement of healthcare professionals and adds a new dimension to clinical studies (34–39). A comparison of this patient-reported outcome study and the non-interventional study with MPPT (33) finds that they both reported almost identical outcomes of treatment, i.e., 100% closure rates for acute and chronic pressure ulcers and control of soft tissue infection caused by osteomyelitis. They also both found that MPPT is suitable for self-care with the survey reporting that 83% of the respondents did not depend upon nurse support for treating their wounds or draining fistulas. This confirms the potential of MPPT for freeing up substantial nursing resources; it has recently been reported that community nurses spend over 50% of their time performing wound dressing changes (40). The high degree of agreement across studies supports the validity of the reported clinical findings.

In conclusion, the survey findings are in agreement with previous clinical evaluations of MPPT in persons with SCI (26, 32, 33). Respondents were overall highly positive towards MPPT and found that MPPT improved their quality-of-life. The healing of pressure ulcers will necessarily also result in substantial cost-savings and freeing up of resources, both in relation to the actual wound but also by avoiding the long list of follow-on conditions if the wound does not heal. The findings, therefore, from both a patient and a health-economic perspective, demonstrate that MPPT can provide significant benefits in healthcare practice.

## Data Availability

The raw data supporting the conclusions of this article will be made available by the authors, without undue reservation.
