# Supplementary material for: Patient-reported outcome survey of user-experiences in the spinal cord injured-community with MPPT for treating wounds and pressure injuries and for controlling soft tissue infection caused by osteomyelitis
Source: Front Rehabil Sci. 2024 Jun 20;5:1386518. doi: 10.3389/fresc.2024.1386518 (PMC11222669; doi:10.3389/fresc.2024.1386518)
Supplement: Supplementary file 1 [file Datasheet1.pdf]

Supplement 1: Wound products used prior to MPPT

Participants were asked "*Did you try any other treatments before using Amicapsil*? (Yes/No); followed by the open-ended question: "*What dressings, treatments did you try, and for how long?*". The responses provided are given below.

BED REST.......TALC
I used Vashe wound wash, Medihoney wound gel, and hydrocolloid dressings.
Medi honey/allevyn dressing
Old wound- inadine
Allevyn. A vacuum dressing which I can't remember the name. Various other dressings.
kerralite cool, manuka honey & various others
Flamminal. Two months
Aquacel + Allevyn for a month
Iodine, clinical plaster for 2 months
Allevyn and Inadine applied by district nurses
Honey
Mepilex border and Alevyn AG at various stages after seeing GP and TVN for over 6 months.
Manuka honey, Omnímatrix cream and aquacel
Medihoney + Mepilex dressing
Multiple types from District nurses
Duoderm dressing, manuka honey, aquagel
savlon
Dakokote and honey med
Mepilex border and other dressings for two weeks
Silver
Pro shield and inadine
Pico, vac, honey, over 4 years
UrgoClean, UrgoStart, Aquacel ribbons, Protosan as a gel, and as a liquid, ribbons with silver, promogran
Tried everything, 15 months under Salisbury TV then home to district nurse
As recommended by District Nurse.
Tried all conventional treatments under care of Spinal Unit, District Nurses and lastly a Tissue Viability Nurse. I had trouble with intolerance for dressing adhesive. Have identified it as acrylate which is in

most dressings. I would use a dressing fir a few days and then have to stop to let the dermatitis clear up
Aquacel primary and Allevyn secondary
spinal injury recommended a bandage which made is worse and infected
Vac dressings
Zinc, urgu clean, seaweed
I have used every dressing available from the NHS over the 12 years
Packing the wound several times a week at local GP surgery, various dressings and Vac machines - over the course of a year and a half.
Alevyn..Vacs,Picos,lava therapy, honey, silver,aqua cell ag, aqua cell, flaminol, Seaweed dressing, iodine dressing, tegaderm dressing, aqua cell extra, kliniderm dressing, Prontosan wash, invading,for the past 6-7 yrs before starting Amicapsil in December 2021
Various dressings and packing of deep wound. Cleansed with Prontosan which has been associated with excessive cytotoxicity
Every type of wound care that the NHS district nurses offer.
